# Asbestos Induces Oxidative Stress and Activation of Nrf2 Signaling in Murine Macrophages: Chemopreventive Role of the Synthetic Lignan Secoisolariciresinol Diglucoside (LGM2605)

**DOI:** 10.3390/ijms17030322

**Published:** 2016-03-01

**Authors:** Ralph A. Pietrofesa, Anastasia Velalopoulou, Steven M. Albelda, Melpo Christofidou-Solomidou

**Affiliations:** Division of Pulmonary, Allergy, and Critical Care Medicine and the Department of Medicine, University of Pennsylvania Perelman School of Medicine, 3615 Civic Center Boulevard, Abramson Research Center, Office Suite 1016C, Philadelphia, PA 19104, USA; ralphp@mail.med.upenn.edu (R.A.P.); avela@mail.med.upenn.edu (A.V.); albelda@mail.med.upenn.edu (S.M.A.)

**Keywords:** antioxidant, asbestos, LGM2605, lignan, macrophage, mesothelioma, oxidative stress, phase II enzymes, reactive oxygen species, secoisolariciresinol diglucoside

## Abstract

The interaction of asbestos fibers with macrophages generates harmful reactive oxygen species (ROS) and subsequent oxidative cell damage that are key processes linked to malignancy. Secoisolariciresinol diglucoside (SDG) is a non-toxic, flaxseed-derived pluripotent compound that has antioxidant properties and may thus function as a chemopreventive agent for asbestos-induced mesothelioma. We thus evaluated synthetic SDG (LGM2605) in asbestos-exposed, elicited murine peritoneal macrophages as an *in vitro* model of tissue phagocytic response to the presence of asbestos in the pleural space. Murine peritoneal macrophages (MFs) were exposed to crocidolite asbestos fibers (20 µg/cm^2^) and evaluated at various times post exposure for cytotoxicity, ROS generation, malondialdehyde (MDA), and levels of 8-iso Prostaglandin F2α (8-isoP). We then evaluated the ability of LGM2605 to mitigate asbestos-induced oxidative stress by administering LGM2605 (50 µM) 4-h prior to asbestos exposure. We observed a significant (*p* < 0.0001), time-dependent increase in asbestos-induced cytotoxicity, ROS generation, and the release of MDA and 8-iso Prostaglandin F2α, markers of lipid peroxidation, which increased linearly over time. LGM2605 treatment significantly (*p* < 0.0001) reduced asbestos-induced cytotoxicity and ROS generation, while decreasing levels of MDA and 8-isoP by 71%–88% and 41%–73%, respectively. Importantly, exposure to asbestos fibers induced cell protective defenses, such as cellular Nrf2 activation and the expression of phase II antioxidant enzymes, HO-1 and Nqo1 that were further enhanced by LGM2605 treatment. LGM2605 boosted antioxidant defenses, as well as reduced asbestos-induced ROS generation and markers of oxidative stress in murine peritoneal macrophages, supporting its possible use as a chemoprevention agent in the development of asbestos-induced malignant mesothelioma.

## 1. Introduction

Malignant mesothelioma (MM) is a highly aggressive cancer that arises from the mesothelial cells of the pleura and peritoneum with a median survival of about one year [1,2,3]. It has now been clearly established in animal models and in patients that asbestos fiber inhalation can lead to neoplastic diseases, such as malignant mesothelioma and lung cancer [4,5], as well as pulmonary fibrosis. Current therapies, other than surgery in early disease states, are not curative [6]. MM represents a global health burden, causing approximately 3000 deaths per year in the United States and an additional 5000 deaths per year in Western Europe. Although asbestos use has been restricted in many Western countries, it is still used in many countries around the world and it is estimated that more than two million tons were mined in 2008. Due to the long latency period of MM development (often up to 30–50 years), and the continued exposure to occupational and environmental asbestos worldwide, the future incidence of MM will likely increase.

Persistent asbestos fibers cause the generation of reactive oxygen species (ROS) and oxidative tissue damage that are implicated in the pathogenesis of asbestos-induced cancers [4,5]. Inhaled asbestos fibers permeate into the lung, and ultimately to the pleural surface, where they are taken up by tissue phagocytes, primarily macrophages [7,8]. It is hypothesized that macrophages exposed to asbestos then undergo frustrated phagocytosis of elongated fibers [9]. This process generates harmful intracellular ROS leading to DNA damage, genomic instability, and ultimately malignant transformation of mesothelial cells [10,11,12,13,14]. A well-tolerated and safe agent with antioxidant properties could thus potentially be used to prevent the development of malignant mesothelioma (MM) in asbestos-exposed populations in a chemopreventive context. According to Greenwald, “the chemoprevention of cancer aims to prevent, arrest, or reverse either the initiation phase of carcinogenesis or the progression of neoplastic cells to cancer” [15]. There is a current unmet need to develop a chemopreventive agent effective against asbestos-induced mesothelioma in high-risk populations. Considering the poor prognosis associated with malignant mesothelioma [16,17], chemopreventive strategies may prove to be beneficial in preventing or delaying MM development.

Previous studies in various models of oxidative stress and pulmonary disease, including acute lung injury from hyperoxia, acid aspiration sepsis [18], ischemia/reperfusion [19,20] and radiation-induced oxidative tissue damage [21] suggested that the flaxseed lignan, secoisolariciresinol diglucoside (SDG) has these requisite antioxidant and ROS scavenging properties [22,23]. We thus hypothesized that SDG or an SDG-rich flaxseed lignan component (FLC)-rich diet might be useful in the chemoprevention of asbestos-induced malignant mesothelioma and have begun a series of studies to test the validity of this idea. In our first study, we evaluated the usefulness of an FLC-supplemented diet in a murine model of acute asbestos-induced peritoneal inflammation and oxidative tissue damage. Three days after intraperitoneal instillation of asbestos into mice, we observed an increase in oxidative and nitrosative stress in the peritoneal fluid, which was significantly attenuated by FLC diet [24]. These findings have led us to conduct more controlled *in vitro* studies where we can confirm that the effects were due specifically to the purified compound SDG, show that peritoneal macrophages were a key target in this antioxidant effect, and conduct additional mechanistic studies.

To study the chemopreventive usefulness of SDG, in preparation for animal and ultimately human studies, SDG was chemically synthesized (LGM2605) by a proprietary pathway [22]. Similarly to natural SDG extracted from whole grain flaxseed, LGM2605 is a free radical scavenger and antioxidant, with DNA-protective activity [23]. Importantly, in a recent study, LGM2605 showed potent cell-protective and antioxidant properties, while capable of inducing phase II antioxidant, cell-protective enzymes transcriptionally regulated by the nuclear factor (erythroid-derived 2)-like 2 (Nrf2)-antioxidant response element (ARE) signaling pathway [25].

In the present study, we evaluated the usefulness of LGM2605 in preventing asbestos-induced ROS generation and oxidative cell damage in murine peritoneal macrophages. Our objectives were: (1) to characterize the antioxidant response in murine peritoneal macrophages following asbestos exposure; (2) to evaluate the ability of LGM2605 to interfere with asbestos-induced ROS generation and oxidative cell damage in macrophages; and (3) to determine the potential molecular mechanisms through which LGM2605 exerts antioxidant effects following asbestos exposure.

## 2. Results

To determine the usefulness of synthetic SDG (LGM2605) in preventing asbestos-induced ROS generation and oxidative cell damage, we utilized elicited murine peritoneal macrophages as a model of tissue phagocytic response to the presence of asbestos in the pleural space. We determined the kinetics of asbestos-induced ROS generation, cytotoxicity, oxidative cell damage, Nrf2 activation, and associated changes in relevant Nrf2-regulated phase II antioxidant enzymes at various times post asbestos exposure.

### 2.1. Determination of Dose-Response Following Asbestos Exposure and LGM2605

**Figure 1:**
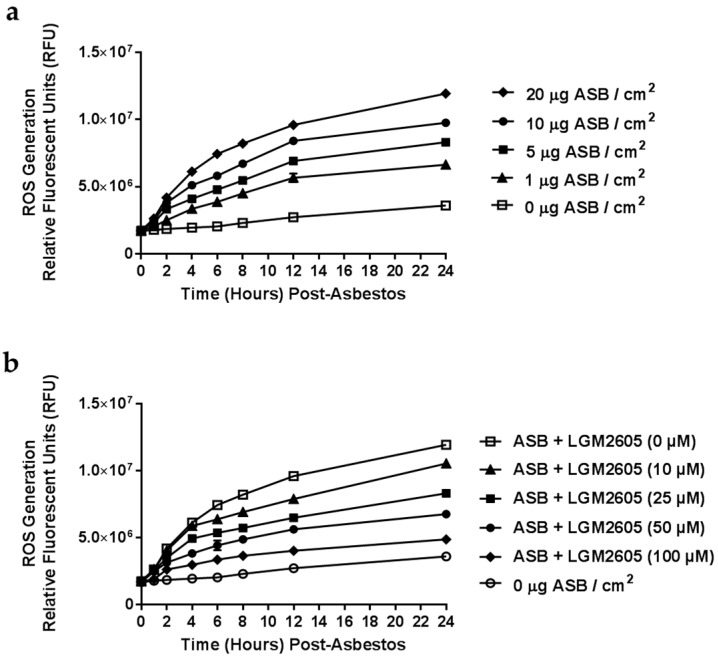
Determination of Dose-Response Following Asbestos Exposure and LGM2605. (**a**) Elicited murine peritoneal macrophages were plated in a 96-well plate (2 × 10^4^ cells/well) and exposed to various concentrations of sterile, standard UICC (Union Internationale Contre le Cancer) crocidolite (SPI Supplies, West Chester, PA, USA) asbestos fibers (0, 1, 5, 10, and 20 µg/cm^2^) to first assess the dose-dependent increase in asbestos-induced ROS generation; (**b**) Various concentrations (0, 10, 25, 50, and 100 µM) of synthetic SDG (LGM2605) were administered 4-h prior to exposure to crocidolite asbestos (20 µg/cm^2^). Culture medium was evaluated for ROS generation using the cell-permeant fluorescent probe 2′,7′-dichlorodihydrofluorescein diacetate (H2DCFDA).

We first empirically determined the optimal level of asbestos exposure (Figure 1a) and LGM2605 dosing (Figure 1b) in murine elicited peritoneal macrophages. We evaluated asbestos-induced ROS generation following cell exposure to various concentrations of crocidolite asbestos fibers (0, 1, 5, 10, and 20 µg/cm^2^). Macrophage exposure to crocidolite asbestos fibers led to significant ROS generation in a dose-dependent manner (Figure 1a). The 20 µg/cm^2^ dose of asbestos induced a robust 2.3-fold increase in ROS and was thus selected as the dose for all experiments performed. Additionally, we tested the antioxidant properties of LGM2605 at various concentrations (0, 10, 25, 50, and 100 µM) and determined a significant reduction in ROS following asbestos exposure in a dose-responsive fashion (Figure 1b). Treatment with 50 µM LGM2605 led to a significant 43% reduction in ROS relative to untreated macrophages and was selected as the optimal treatment dose for evaluating the preventive properties of LGM2605 in attenuating the deleterious effects of asbestos exposure. Importantly, the dose of LGM2605 (µM) used in the *in vitro* studies described here is comparable to tissue levels achieved following oral administration of a physiologically relevant dose in mice.

### 2.2. LGM2605 Reduces Asbestos-Induced ROS Generation and Cytotoxicity

**Figure 2:**
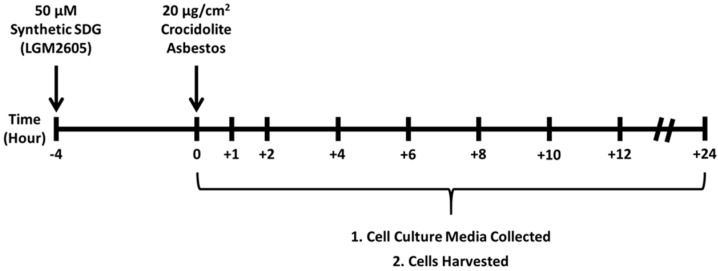
Experimental plan of asbestos exposure, LGM2605 Treatment, and Harvest Time Points. Murine peritoneal macrophages were exposed to 50 µM synthetic SDG (LGM2605) 4-h prior to exposure to sterile UICC crocidolite (SPI Supplies) asbestos fibers (20 µg/cm^2^). Culture medium and cells were harvested at 0, 1, 2, 4, 6, 8, 12, and 24-h post asbestos exposure. Culture medium was evaluated for H_2_O_2_ content, lactate dehydrogenase (LDH) release, levels of MDA and 8-iso Prostaglandin F2a, while cells were harvested for gene and protein expression analysis.

We next utilized elicited murine peritoneal macrophages to study the effect of LGM2605 treatment on asbestos-induced cellular ROS generation, oxidative stress, and induction of Nrf2 signaling. LGM2605 treatment (50 µM) was initiated 4-h prior to exposure to crocidolite asbestos fibers (20 µg/cm^2^) and cell culture medium and macrophages were collected at 0, 1, 2, 4, 6, 8, 12, and 24-h post asbestos exposure (Figure 2).

**Figure 3:**
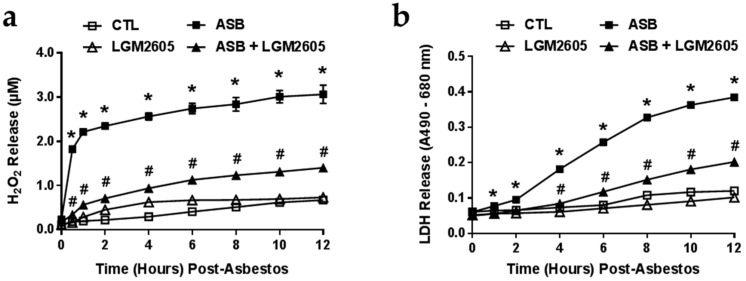
ROS Production and Cytotoxicity Following Asbestos Exposure. Elicited murine peritoneal macrophages were plated in a 6-well plate (2 × 10^6^ cells/well) and exposed to 50 µM synthetic SDG (LGM2605) 4-h prior to exposure to sterile UICC crocidolite (SPI Supplies) asbestos fibers (20 µg/cm^2^). (**a**) Murine peritoneal macrophage release of H_2_O_2_ (**b**) and lactate dehydrogenase (LDH) was determined at 0, 1, 2, 4, 6, 8, 10 and 12-h post asbestos exposure. Samples were run undiluted in triplicate and data are presented as mean ± the standard error of the mean (SEM). * indicates a statistically significant difference *(p* < 0.05) between asbestos-only (ASB) and control (CTL) treated cells; ^#^ indicates a statistically significant difference (*p* < 0.05) between ASB and asbestos + LGM2605 (ASB + LGM2605) treated cells.

We determined the kinetics of ROS generation and cytotoxicity following exposure to 20 µg/cm^2^ crocidolite asbestos by measuring levels of H_2_O_2_ released into the culture medium a*s* measured by Amplex Red (Figure 3a). Minimal H_2_O_2_ was released by control (non-asbestos-exposed) cells or by cells treated with LMG2605-alone. In contrast, H_2_O_2_ levels rapidly increased within 30 min post asbestos exposure (from 0.24 ± 0.03 to 1.83 ± 0.06 µM H_2_O_2_) and remained elevated after 12-h. Pretreatment with LGM2605 at a dose of 50 µM significantly (*p* < 0.0001) reduced levels of H_2_O_2_ by 54%–80% (Figure 3a). We conducted similar studies to assess asbestos-induced cytotoxicity by measuring LDH release (Figure 3b). Again, minimal LDH was released by control (non-asbestos-exposed) cells or by cells treated with LMG2605-alone. However, starting at 4-h after asbestos exposure (following the initial increase in asbestos-induced H_2_O_2_ release), we detected a significant (*p* < 0.0001) cytotoxic effect. Pretreatment with LGM2605 at a dose of 50 µM significantly (*p* < 0.0001) reduced LDH release (Figure 3b). Levels of H_2_O_2_ and LDH released into the cell culture medium were not determined 24-h post asbestos exposure, since the release of H_2_O_2_ and LDH into the cell culture medium had reached a plateau by 10 to 12-h post asbestos exposure.

### 2.3. The Synthetic SDG (LGM2605) Reduces Asbestos-Induced Lipid Peroxidation and Oxidative Cell Damage

**Figure 4:**
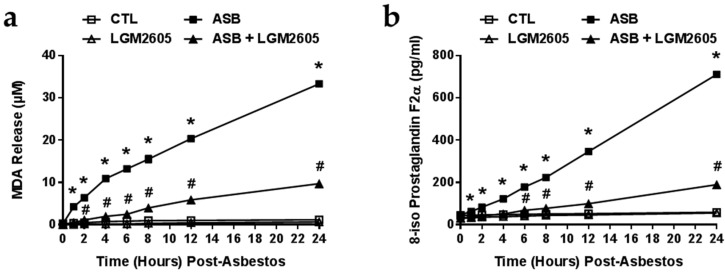
LGM2605 Prevents Oxidative Cell Damage and the Release of MDA and 8-IsoP. Elicited murine peritoneal macrophages were plated in a 6-well plate (2 × 10^6^ cells/well) and exposed to 50 µM synthetic SDG (LGM2605) 4-h prior to exposure to sterile UICC crocidolite (SPI Supplies) asbestos fibers (20 µg/cm^2^). The levels of (**a**) MDA and (**b**) 8-iso Prostaglandin F2a were determined in the cell culture medium at 0, 1, 2, 4, 6, 8, 12 and 24-h post asbestos exposure. Data are presented as mean ± SEM. * indicates a statistically significant difference (*p* < 0.05) between ASB and CTL treated cells; ^#^ indicates a statistically significant difference (*p* < 0.05) between ASB and ASB + LGM2605 treated cells.

Minimal levels of MDA were detected in control (non-asbestos-exposed) cells or by cells treated with LMG2605-alone (Figure 4a). Levels of MDA were significantly (*p* < 0.0001) elevated after 30-min of asbestos exposure and continued to accumulate in the cell culture medium (33.16 ± 0.58 µM at 24-h post asbestos). LGM2605 treatment significantly (*p* < 0.0001) reduced levels of MDA by 71%–88% (9.70 ± 0.54 µM at 24-h post asbestos) (Figure 4a).

We also evaluated levels of 8-iso Prostaglandin F2a in the cell culture medium as a marker of oxidative stress and cell damage following asbestos exposure (Figure 4b). Minimal levels of 8-isoP were released by control (non-asbestos-exposed) cells or by cells treated with LMG2605-alone. Asbestos exposure led to a significant increase in the concentration of 8-isoPs (710.87 ± 5.55 pg/mL at 24-h post asbestos) that was significantly blunted by the administration of LGM2605 (189.89 ± 2.58 pg/mL) (Figure 4b).

### 2.4. Exposure to Crocidolite Asbestos Fibers Induces Phase II Antioxidant Enzyme Expression in Murine Macrophages

**Figure 5:**
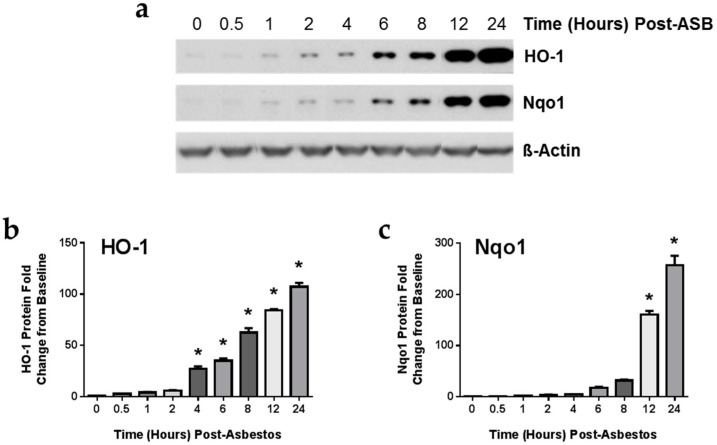
Asbestos Exposure Induces the Expression of Phase II Antioxidant Enzymes. Elicited murine peritoneal macrophages were harvested after 0, 0.5, 1, 2, 4, 5, 8, 12, and 24-h of asbestos exposure and evaluated by (**a**) Western blotting for HO-1 and Nqo1. Densitometric analysis of band intensity for (**b**) HO-1 and (**c**) Nqo1 was normalized to β-actin and values are expressed as fold change from CTL at time 0. Data are presented as mean ± SEM. * indicates a statistically significant (*p* < 0.05) increase in HO-1 and Nqo1 protein levels above baseline values at time 0.

To investigate the underlying mechanisms behind the decrease in lipid peroxidation and oxidative stress by LGM2605 treatment, we evaluated protein and gene expression changes in heme oxygenase-1 (HO-1) and NADPH: quinone oxidoreductase-1 (Nqo1) (Figure 5), important enzymes stimulated by the Nrf2-ARE pathway critical in the detoxification of free radicals and reactive oxygen species. We first determined the kinetics of asbestos-induced protein expression of HO-1 and Nqo1. Starting at a very low baseline, asbestos exposure induced the expression of HO-1 (Figure 5a,b) significantly (*p* < 0.05) above baseline levels as early as 4-h post exposure. Levels of Nqo1 increased sharply after 12 h (Figure 5a,c). We therefore evaluated the effect of LGM2605 treatment on antioxidant enzyme gene expression at 8 and 24-h post asbestos exposure.

### 2.5. LGM2605 Enhances Asbestos-Induced Activatation Nrf2 Signaling and Gene Expression of Phase II Enzymes

After evaluating the kinetics of antioxidant enzyme expression following asbestos exposure, we determined the effects of asbestos exposure on Nrf2 signaling. Cellular oxidative stress leads to the activation of the Nrf2-ARE pathway, which transcriptionally regulates many antioxidant and cytoprotective genes responsible for cellular homeostasis. We, thus, determined Nrf2 activation following asbestos exposure and assessed gene expression changes in HO-1 and NQO1.

**Figure 6:**
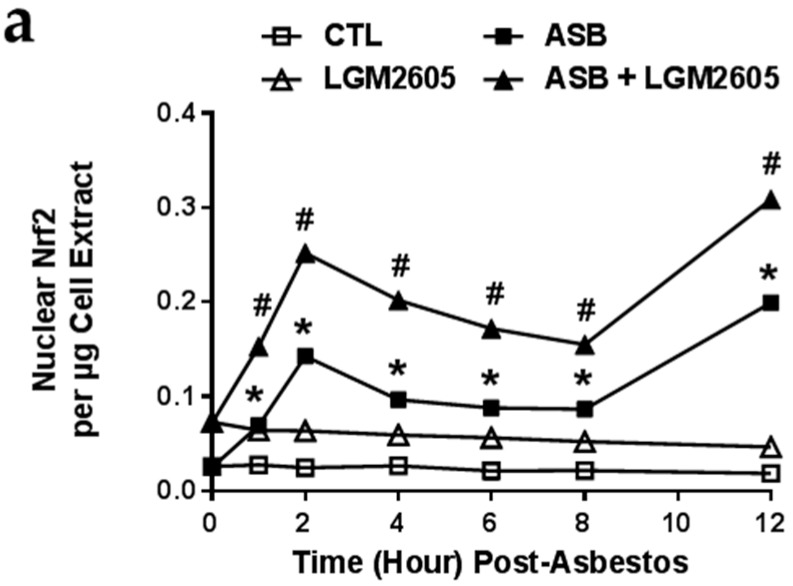

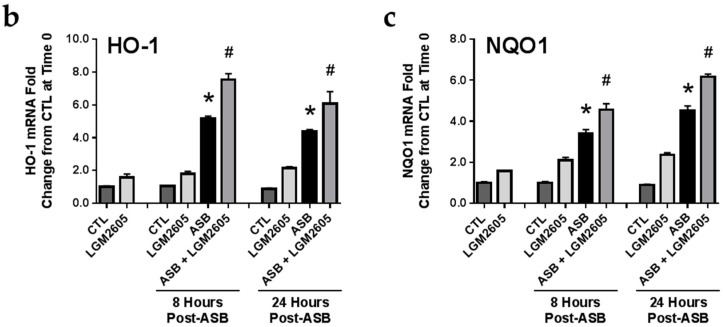
LGM2605 Boosts Nrf2 Activation and the Expression of Antioxidant Enzymes in Elicited Murine Peritoneal Macrophages. (**a**) Levels of active, nuclear Nrf2 were determined at 0, 1, 2, 4, 6, 8, and 12-h post asbestos exposure; Macrophage mRNA expression of (**b**) HO-1 and (**c**) NQO1 was determined at 0, 8, and 24-h post asbestos exposure using qPCR. Levels of target gene mRNA were normalized to β-actin RNA and values are expressed as fold change from CTL. Data are presented as mean ± SEM. * indicates a statistically significant difference (*p* < 0.05) between ASB and CTL treated cells; ^#^ indicates a statistically significant difference (*p* < 0.05) between ASB and ASB + LGM2605 treated cells.

We first determined levels of Nrf2 in macrophage nuclear extracts, an indicator of active Nrf2 that has exited the cytosol after being cleaved from Kelch-like ECH-associated protein 1 (Keap1), and determined the kinetics of Nrf2 nuclear accumulation following asbestos exposure (Figure 6a). Nuclear Nrf2 was low at baseline and did not significantly change over time. However, pretreatment with 50 µM LGM2605 led to significant (*p* < 0.0001) activation of Nrf2 signaling at baseline prior to asbestos exposure that remained elevated through the 12-h of observation (elevated 2.81-fold from control, non-asbestos exposed macrophages). Following asbestos exposure, we observed significantly (*p* < 0.0001) increased levels of nuclear Nrf2, with the highest concentration occurring after 2 and 12-h post exposure. Levels of Nrf2 from nuclear extracts of macrophages treated with LGM2605 and asbestos were significantly (*p* < 0.0001) elevated above asbestos-only exposure.

Upon determination of HO-1 and NQO1 gene expression, treatment with LGM2605-alone led to significant (*p* < 0.0001) elevations in mRNA levels (1.58 ± 0.21 and 1.58 ± 0.02 fold change from control, respectively) at all time points evaluated (Figure 6b,c). Importantly, although mRNA levels of HO-1 and NQO1 were significantly elevated above control after 8 and 24-h of asbestos exposure, treatment with LGM2605 further boosted antioxidant gene expression above asbestos-only exposure (Figure 6b,c) by an additional 28%–31% and 25%–26%, respectively.

### 2.6. Induction of Cellular Antioxidant Enzymes by Asbestos and Further Activation by LGM2605

**Figure 7:**
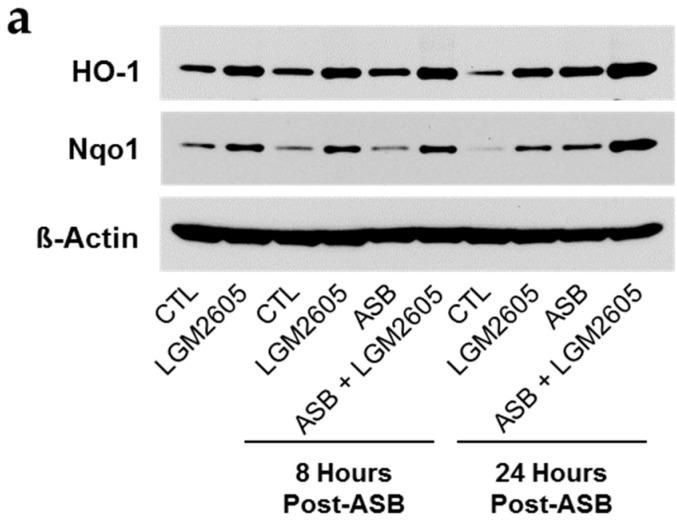

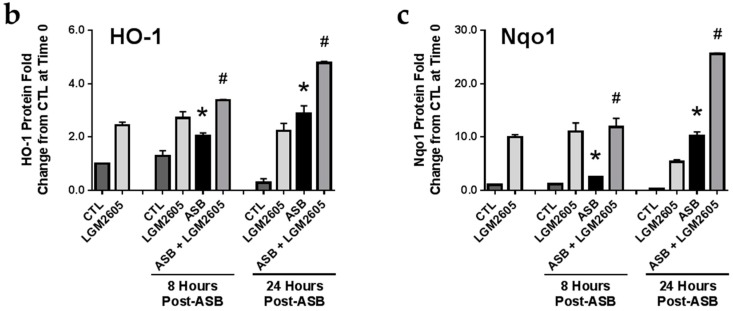
The Synthetic Lignan SDG (LGM2605) Enhances the Expression of Nrf2-Regulated Antioxidant Enzymes in Elicited Murine Peritoneal Macrophages. Levels of antioxidant enzymes were determined by (**a**) Western blotting for HO-1 and Nqo1; Densitometric analysis of band intensity for (**b**) HO-1 and (**c**) Nqo1 was normalized to β-actin and values are expressed as fold change from CTL at time 0. Data are presented as mean ± SEM. * indicates a statistically significant difference (*p* < 0.05) between ASB and CTL treated cells; ^#^ indicates a statistically significant difference (*p* < 0.05) between ASB and ASB + LGM2605 treated cells.

When we evaluated protein levels of these Nrf2-inducible antioxidant enzymes, we observed increased protein expression in macrophages treated with asbestos and LGM2605 at both 8 and 24-h post asbestos exposure. Importantly, treatment of macrophages with LGM2605-alone led to a robust increase in the expression of both HO-1 and Nqo1 prior to asbestos exposure (2.45 ± 0.11 and 9.94 ± 0.49 fold change from control, respectively). Although protein levels of HO-1 (Figure 7a,b) and Nqo1 (Figure 7a,c) were significantly elevated above control after 8 and 24-h of exposure, macrophages treated with LGM2605 displayed a further increase in both antioxidant enzymes by 39%–40% and 60%–79%, respectively.

## 3. Discussion

Our current findings highlight the dual role of LGM2605 in scavenging ROS and inducing phase II antioxidant enzymes in an *in vitro* model of asbestos exposure utilizing murine peritoneal macrophages as an *in vitro* model of tissue phagocytic response to the presence of asbestos in the pleural space. First, we observed the ability of LGM2605 to blunt asbestos-induced ROS generation and cytotoxicity. Lipid peroxidation plays a major role in mediating oxidative damage in tissues, is a qualitative indicator of oxidative stress within tissues and cells, and can be measured by determining the amount of malondialdehyde (MDA), a product of lipid peroxidation [21,26]. We determined decreased levels of MDA and 8-isoP with LGM2605 treatment, indicative of decreased asbestos-induced oxidative stress. Importantly, while asbestos exposure activates Nrf2 signaling and the expression of phase II antioxidant enzymes, LGM2605 further enhances levels of key antioxidant enzymes involved in the detoxification of reactive oxygen species. Of note, LGM2605 boosted antioxidant defenses at baseline prior to asbestos exposure and were further augmented by the asbestos challenge. Our findings support the usefulness of LGM2605 as a potential chemopreventive agent in reducing the early cytotoxicity and oxidative cell damage that occurs following asbestos exposure.

We have previously evaluated the ability of a flaxseed lignan diet, enriched in the natural form of biphenolic SDG, to prevent acute asbestos-induced inflammation and oxidative/nitrosative stress in a murine model of peritoneal asbestos exposure [24]. Specifically, mice fed an FLC diet, enriched in SDG, displayed a 74.6% reduction in levels of the MDA in the peritoneal lavage fluid (PLF) at three days post intraperitoneal asbestos exposure. In accordance with our current findings, peritoneal white blood cells from mice fed an SDG-rich FLC diet had significantly increased mRNA levels of HO-1, NQO1, and glutathione *S*-transferase mu 1 (GSTM1) compared to CTL-fed mice. Combined, our findings highlight the usefulness of SDG (LGM2605) in the detoxification of free radicals and ROS following asbestos exposure. Importantly, our current findings point towards the role of LGM2605 as an activator of Nrf2 signaling and an inducer of phase II antioxidant enzymes. In addition, we have confirmed that the antioxidant effects from feeding an SDG-rich FLC diet were due specifically to the purified compound SDG (LGM2605) and have shown that peritoneal macrophages are a key target in this antioxidant effect.

Although the idea of chemoprevention sounds simple, it has been very difficult to find effective cancer chemopreventive agents. First, the mechanisms by which carcinogens induce cancer usually involve multiple molecular pathways, making efficacy challenging and requiring an agent with multiple biological activities. Second, since the agent will be used to prevent a small number of tumors in a large population of healthy, but at-risk individuals, it must be extraordinarily non-toxic, well-tolerated, and affordable. Dietary chemoprevention strategies for induction of Nrf2/ARE activation have been attempted in multiple studies [27] using dietary natural or synthetic agents such as isothiocyanates or dithiolethiones [28,29]. The cancer chemopreventive actions of Oltipraz (OLZ), a member of a class of 1,2-dithiolethiones, primarily associated with the induction of phase II enzymes was shown in studies to decrease tumor burden in mice in an Nrf2-dependent manner. OLZ, like most other known phase II inducers, was associated with serious and deleterious adverse effects in human clinical trials, including hepatotoxicity [30]. Agents such as OLZ are actually oxidants, themselves, that stimulate Nrf2 by placing the cell under mild oxidant stress [31]. Specifically, dithiolethiones were recently found to generate superoxide, thus providing a mechanistic explanation for their ability to induce Nrf2-dependent phase II enzymes [32]. Unfortunately, these agents have also proven to be rather toxic [33]. Flaxseed lignans in contrast, directly detoxify ROS, specifically the hydroxyl radical (•OH), which is the most reactive of all reactive oxygen species [34,35]. A particularly exciting aspect of our findings is that flaxseed or the flaxseed lignan SDG and its synthetic counterpart, LGM2605, all activate the Nrf2-ARE system, thus amplifying its antioxidant potency, but in a uniquely non-toxic fashion. Safe, non-toxic use of flaxseed or its lignan components was shown not only by our group [19,25,36], but has been confirmed by others as well [37,38,39].

SDG, an antioxidant isolated from flaxseed, is metabolized by intestinal bacteria to enterodiol (ED), and enterolactone (EL) which are bioactive. However, SDG also has strong direct antioxidant properties *in vitro* without the need for metabolic activation [21]. The antioxidant activities of these three lignans (SDG, EL and ED) were shown by their ability to inhibit linoleic acid lipid peroxidation, indicating direct hydroxyl radical scavenging activity [35,40]. Since oxidant stress is implicated in the etiology of cancer, the therapeutic or preventive use of dietary flaxseed or flaxseed-derived lignans has been considered in some tumors. Lignans were shown to reduce chemically-induced mammary and colon tumorigenesis in rats [41,42,43] and experimental metastasis of melanoma cells in mice [44]. The flaxseed lignan SDG is now emerging as a potential anticarcinogenic agent and found to modulate growth factor-mediated cell signaling, cell cycle gene expression and apoptosis [45,46,47,48].

Flaxseed or flaxseed lignans have never been evaluated in lung diseases until our group pioneered its use in a series of studies including hyperoxia, acid aspiration, and lipopolysaccharide- induced acute lung injury [18]. We recently extended the findings of flaxseed protection in models of warm lung ischemia/reperfusion injury [19] and pneumonitis, resulting from thoracic radiation [21]. In the course of our studies, we made the important observation that flaxseed, specifically the bioactive lignan component enriched in SDG, had the ability to upregulate phase II enzymes and other antioxidant enzymes in cell culture and in whole animals through activation of the transcription factor Nrf2 [25,49]. The ability of flaxseed to activate Nrf2, coupled with its direct antioxidant activity generated our key hypothesis, that flaxseed could function as a safe, non-toxic chemopreventive agent for asbestos-induced mesothelioma.

Asbestos fibers have been shown to participate in redox reactions to generate reactive oxygen species by multiple mechanisms, including hydroxyl radicals generated either through a redox reaction or by catalyzing a Fenton-like reaction in exposed cells [50]. Asbestos fiber internalization generates a significant increase in intracellular ROS and there is considerable evidence that asbestos-initiated chronic oxidative stress contributes to carcinogenesis and fibrosis by promoting oxidative DNA damage and regulating redox signaling pathways in exposed cells [51].

We report the ability of LGM2605 to both scavenge free radicals and detoxify ROS through direct and indirect molecular effects. We have previously reported the direct free radical scavenging ability of SDG in a murine endothelial cell model of gamma radiation-induced ROS [21] and therefore studied this potential mechanism in our system. LGM2605 likely acted as a direct free radical scavenger and antioxidant in a dose-responsive manner. In addition to the direct free radical scavenging ability of LGM2605, we have shown that flaxseed, and it bioactive lignan component, can activate Nrf2 in normal cells and tissues [18,19,21,49,52]. Nrf2 is a master transcriptional regulator of carcinogen detoxifying and antioxidant enzymes (such as HO1 and Nqo1) and plays a major role in tissue protection. These findings are in agreement with those of Velalopoulou *et al.* [25], where LGM2605 protected normal lung cells from radiation-induced DNA damage through direct free radical scavenging and boosting of endogenous antioxidant enzyme gene expression.

In a recent review Neri *et al.* [5] summarized the status of chemoprevention of asbestos-linked cancer. Almost all of the 46 citations focused on aspects of a series of large clinical trials started between 1985 and 1991, using one agent: β-carotene. This was based on observational studies showing that people eating more fruits and vegetables, which are rich in β-carotene, had lower rates of lung cancer. They identified three large published trials: the CARET trial (which had 4000 asbestos-exposed workers), the Tyler Asbestos Workers Program (755 exposed persons), and the Wittenoom Crocidolite Industry Program (~1000 participants). Unfortunately, the results in all of these trials showed either an increased incidence of cancer or no clear efficacy. No new trials have been started in the last 20 years. In their review, Neri *et al.* [5] only found three publications testing other agents in *in vitro* studies looking at an inhibitor of cyclooxygenase-2 (COX-2), selenium, and butyrate. Two recent additional studies evaluated the effects of a COX-2 inhibitor and of Vitamin A, Vitamin E, and selenium in a mouse model of asbestos-induced MM [53,54]. These studies used a new transgenic mouse model of mesothelioma in which the SV40 large T-antigen was driven by a mesothelin promoter (MexTag mice) [53]. These mice do not spontaneously develop MM, but if injected with asbestos intraperitoneally will all reproducibly develop mesothelioma within 40 weeks. Of note, none of the agents tested reduced the incidence of asbestos-induced MM.

Despite past failures [5] and considerable challenges in identifying a useful chemopreventive agent, we believe the strategy of chemoprevention for asbestos-induced MM holds special promise and should be actively pursued because: (1) we have the ability to identify a clear at-risk population (asbestos-exposed individuals); (2) there is a long latency period where interventions will have time to work; (3) there is no effective therapy or proven screening approach; (4) good mouse models exist which closely mimic the human situation where asbestos causes MM; (5) there is a new and growing understanding of the mechanisms by which asbestos induces MM; (6) some interesting new biomarkers are emerging, *i.e.*, HMGB1 [55], mesothelin [56], and fibulin-3 [57] that could provide key intermediate endpoints, and (7) we have identified what we think is an ideal non-toxic, yet potentially efficacious, chemopreventive agent (LGM2605) that inhibits both asbestos-induced inflammation and ROS/reactive nitrogen species (RNS) generation.

## 4. Materials and Methods

### 4.1. Harvesting of Murine Peritoneal Macrophages

Murine peritoneal macrophages (MF) were harvested from the peritoneum following elicitation using thioglycollate broth. Mice were used at 13 weeks of age under animal protocols approved by the Institutional Animal Care and Use Committee (IACUC) of the University of Pennsylvania, (Philadelphia, PA, USA). Animals were housed in conventional cages under standardized conditions with controlled temperature and humidity, and a 12–12-h day-night light cycle. Animals had free access to water and mouse chow. Mice were injected, via intraperitoneal (IP) injection, with 1 mL of a 3% solution of thioglycollate broth in 0.5 mL phosphate-buffered saline (PBS). Three days following thioglycollate exposure, mice were euthanized using an overdose of ketamine (160 mg/kg) and xylazine (25 mg/kg). Peritoneal lavage (PL) was then performed through a 20-gauge angiocatheter (BD Pharmingen, San Diego, CA, USA), with the intraperitoneal instillation of 3 mL Hanks balanced salt solution (HBSS; Ca^2+^ and Mg^2+^ free). An aliquot of peritoneal lavage fluid (PLF) was immediately separated to measure total white blood cell (WBC) counts (cells/ml PLF) using a Coulter Cell and Particle Counter (Beckman Coulter, Miami, FL, USA). Murine peritoneal macrophages were plated in 1 mL of cell culture medium phenol-free RPMI supplemented with 1% FBS, penicillin (100 units/mL) and streptomycin (100 µg/mL), and l-Glutamine (2 mm) in a 6-well plate (2 × 10^6^ cells/well) and allowed to adhere to the bottom of the wells. Elicited peritoneal macrophages were used to determine the effects of LGM2605 in preventing asbestos-induced cytotoxicity, generation of reactive oxygen species (ROS), oxidative cell damage, and cellular antioxidant response.

### 4.2. Crocidolite Asbestos Exposure

Elicited peritoneal macrophages were exposed to sterile UICC (Union Internationale Contre le Cancer) crocidolite (Na_2_O·Fe_2_O_3_·8SiO_2_·H_2_O) asbestos fibers (SPI Supplies, West Chester, PA, USA) that were baked overnight, resuspended in 1X PBS at a stock concentration of 800 µg/mL and sonicated for 30 min. The solution of asbestos fibers was exposed to ultraviolet light prior to use in cell culture experiments. Initial experiments were conducted using various concentrations of asbestos fibers (0, 1, 5, 10, and 20 µg/cm^2^) to assess the dose-dependent effect of asbestos exposure (see Figure 1a). In all subsequent experiments, murine peritoneal macrophages were exposed to crocidolite asbestos fibers at a concentration of 20 µg/cm^2^.

### 4.3. Synthetic SDG (LGM2605) Exposure

Synthesis of secoisolariciresinol diglucoside has been previously described [22]. Briefly, secoisolariciresinol diglucosides (*S*,*S*)-SDG (the major isomer in whole grain flaxseed) and (*R*,*R*)-SDG (the minor isomer in whole grain flaxseed) were synthesized from vanillin via secoisolariciresinol and a glucosyl donor (perbenzoyl-protected trichloroacetimidate under the influence of TMSOTf) through a concise route that involved chromatographic separation of diastereomeric diglucoside derivatives. Synthetic SDG (LGM2605) was reconstituted to a stock concentration of 10 mM and cells were exposed to 50 µM SDG 4-h prior to asbestos exposure (see Figure 2). Initial experiments were conducted using various concentrations of LGM2605 (0, 10, 25, 50, and 100 µM) to assess the dose-dependent effect of LGM2605 treatment (see Figure 1b). In all subsequent experiments, murine peritoneal macrophages were treated with 50 µM LGM2605 4-h prior to asbestos exposure.

### 4.4. Determination of Intracellular Asbestos-Induced ROS Generation

Levels of intracellular ROS were determined using the cell-permeant fluorescent probe 2′,7′-dichlorodihydrofluorescein diacetate (H2DCFDA) (Molecular Probes^®^, ThermoFisher Scientific, Waltham, MA, USA). Upon cleavage of the acetate groups by intracellular esterases and oxidation, the nonfluorescent H2DCFDA is converted to the highly fluorescent 2′,7′-dichlorofluorescein (DCF). Elicited murine peritoneal macrophages were plated in a 96-well plate (2 × 10^4^ cells/well) and exposed to various concentrations of sterile UICC crocidolite (SPI Supplies) asbestos fibers (0, 1, 5, 10, and 20 µg/cm^2^) and LGM2605 (0, 10, 25, 50, and 100 µM). The fluorescence intensity was then measured on a SpectraMax i3x Multi-Mode microplate reader (Molecular Devices, Sunnyvale, CA, USA) using an excitation wavelength in the range of 492–495 nm and a fluorescence emission detection at 517–527 nm.

### 4.5. Quantification of H_2_O_2_ Release from Peritoneal Macrophages Following Asbestos Exposure

As a measure of asbestos-induced ROS generation, we determined extracellular levels of H_2_O_2_ following asbestos exposure at 0, 0.5, 1, 2, 4, 6, 8, 10, and 12-h post-exposure. Elicited murine peritoneal macrophages were plated in a 6-well plate (2 × 10^6^ cells/well) and exposed to 50 µM synthetic SDG (LGM2605) 4-h prior to exposure to sterile UICC crocidolite (SPI Supplies) asbestos fibers (20 µg/cm^2^). The release of H_2_O_2_ by murine peritoneal macrophages was quantified using the Amplex Red enzyme assay (Molecular Probes^®^, ThermoFisher Scientific, Waltham, MA, USA), which utilizes horseradish peroxidase (HRP)-dependent oxidation of *N*-acetyl-3,7-dihydroxyphenoxazine. The fluorescence intensity was then measured on a SpectraMax i3x Multi-Mode microplate reader (Molecular Devices, Sunnyvale, CA, USA) using an excitation wavelength in the range of 530–560 nm and a fluorescence emission detection at 590 nm.

### 4.6. Determination of Asbestos-Induced Cytotoxicity

Asbestos-induced cytotoxicity was determined by quantitatively measuring extracellular levels of lactate dehydrogenase (LDH) released into the cell culture medium. LDH levels were determined using a colorimetric assay (Pierce™ LDH Cytotoxicity Assay Kit, ThermoFisher Scientific, Waltham, MA, USA) according to the manufacturer’s protocol. Briefly, 50 μL of cell culture media was collected at 0, 1, 2, 4, 6, 8, 10, and 12-h post-asbestos exposure and transferred to a 96-well plate. The cell culture medium was incubated with 50 µL of reaction mixture, containing lactate, NAD^+^, diaphorase, and a tetrazolium salt. LDH conversion of lactate to pyruvate generates NADH, which is used by diaphorase to convert the tetrazolium salt to a red formazan product that was measured at 490 nm. Data are reported as LDH cytotoxicity (absorbance 490–680 nm).

### 4.7. Evaluation of Lipid Peroxidation

Malondialdehyde (MDA), an indicator of oxidative stress (19) was measured in the cell culture medium using a commercially available kit (TBARS Assay Kit, Cayman Chemical, Ann Arbor, MI, USA) according to the manufacturer’s protocol. Elicited murine peritoneal macrophages were plated in a 6-well plate (2 × 10^6^ cells/well) and exposed to 50 µM synthetic SDG (LGM2605) 4-h prior to exposure to sterile UICC crocidolite (SPI Supplies, West Chester, PA, USA) asbestos fibers (20 µg/cm^2^). The levels of MDA were determined in the cell culture medium at 0, 1, 2, 4, 6, 8, 12 and 24-h post asbestos exposure. Specifically, levels of thiobarbituric acid reactive substances (TBARS) were quantified by measuring the fluorescence of malondialdehyde-thiobarbituric acid (MDA-TBA) adducts in cell culture medium samples. According to manufacturer instructions, MDA-TBA adducts were formed via acid hydrolysis at 100 °C and measured fluorometrically using a SpectraMax i3x Multi-Mode microplate reader (Molecular Devices, Sunnyvale, CA, USA) with an excitation wavelength of 530 nm and an emission wavelength of 550 nm. Levels of lipid peroxidation in cell culture medium samples are reported as the concentration (nM) of MDA [58].

### 4.8. Analysis of 8-Iso Prostaglandin F2a Levels in the Cell Culture Medium

Levels of 8-iso Prostaglandin F2a (8-IsoP), metabolites of tissue phospholipid oxidation and a biomarker of oxidative stress and antioxidant deficiency, in cell culture medium were determined using an 8-iso Prostaglandin F2a enzyme-linked immunosorbent assay (ELISA) kit (Cayman Chemical, Ann Arbor, MI, USA) according to the manufacturer’s protocol. Elicited murine peritoneal macrophages were plated in a 6-well plate (2 × 10^6^ cells/well) and exposed to 50 µM synthetic SDG (LGM2605) 4-h prior to exposure to sterile UICC crocidolite (SPI Supplies) asbestos fibers (20 µg/cm^2^). The levels of 8-IsoP were determined in the cell culture medium at 0, 1, 2, 4, 6, 8, 12 and 24-h post asbestos exposure. Cell culture medium samples were run undiluted and the data are reported as the concentration (pg/mL) of 8-iso Prostaglandin F2a in the cell culture medium.

### 4.9. Nrf2 Transcription Factor Analysis

The presence of nuclear factor (erythroid-derived 2)-like 2 (Nrf2) was determined in nuclear extracts isolated from macrophages exposed to asbestos and harvested at 1, 2, 4, 6, 8, and 12-h post asbestos exposure. Cytoplasmic and nuclear extracts were prepared using a commercially available nuclear extraction kit (Cayman Chemical, Ann Arbor, MI, USA). A transcription factor assay kit (Cayman Chemical, Ann Arbor, MI, USA) was used to detect nuclear Nrf2. The assay was performed according to the manufacturer’s protocol. The transcription factor assay kit utilizes a specific double-stranded DNA sequence containing the Nrf2 response element to bind to Nrf2 molecules present in the nuclear fraction. The presence of Nrf2 in the nucleus was detected by ELISA per the manufacturer’s instructions. The data are reported as the ratio of the absorbance at 450 nm to the protein concentration of the nuclear extract (µg).

### 4.10. RNA Isolation and Gene Expression Analysis

Total RNA was isolated from murine peritoneal macrophages using a commercially available kit, RNeasy Plus Mini Kit, supplied by Qiagen (Valencia, CA, USA). Total RNA was quantified using a NanoDrop 2000 apparatus (ThermoFisher Scientific, Waltham, MA, USA). Reverse transcription of RNA to cDNA was then performed on a Veriti^®^ Thermal Cycler using the high capacity RNA to cDNA kit supplied by Applied Biosystems Quantitative Polymerase Chain Reaction (qPCR) and performed using TaqMan^®^ Probe-Based Gene Expression Assays supplied by Applied Biosystems, Life Technologies (Carlsbad, CA, USA). Individual TaqMan gene expression assays were selected for relevant cytoprotective and phase II antioxidant enzymes (heme oxygenase-1 (HO-1), and NADPH: quinone oxidoreductase-1 (NQO1)). Quantitative real-time PCR was performed using 50 ng of cDNA per reaction well on a StepOnePlus™ Real-Time PCR System (Applied Biosystems, Life Technologies, Carlsbad, CA, USA). Gene expression data were normalized to β-actin RNA housekeeping gene and calibrated to the control samples (CTL at time 0) according to the ΔΔ*C*T method as previously described [49].

### 4.11. Western Blot Analysis

Immunoblot analysis of murine peritoneal macrophages at 0, 8, and 24-h post-asbestos exposure was performed as previously described [19,25] using primary antibodies against HO-1 (catalogue # ab52947, abcam, Cambridge, MA, USA) and Nqo1 (catalogue # NBP1-40663, Novus Biologicals, Littleton, CO, USA). Protein levels of HO-1 and Nqo1 were detected using manufacturer recommended dilutions and quantified by densitometric analysis of specific bands (33 kDa for HO-1 and 31 kDa for Nqo1) with β-actin normalization of protein expression using Gel-Pro Analyzer software (Version 6.0, MediaCybernetics, Silver Spring, MD, USA).

### 4.12. Statistical Analysis

All data were analyzed using two-way analysis of variance (ANOVA) to test for the main effects of time and treatment on study outcome measures. Post-tests (Tukey’s multiple comparisons tests) were conducted analyzing significant differences among treatment groups (CTL, LGM2605, ASB, and ASB + LGM2605) within each time point. Statistically significant differences were determined using GraphPad Prism version 6.00 for Windows, GraphPad Software, La Jolla, CA, USA (www.graphpad.com). Results are reported as mean ± the standard error of the mean (SEM). Levels of target gene mRNA are reported as the mean fold change from CTL at time 0 ± SEM. Statistically significant differences were determined at *p*-value of 0.05. Asterisks shown in figures indicate significant differences between asbestos-exposed (ASB) and control (CTL) exposure groups. # shown in figures indicates significant differences between asbestos-exposed (ASB) and asbestos-exposed and LGM2605-treated (ASB + LGM2605) macrophages.

## 5. Conclusions

An ideal agent used for the chemoprevention of asbestos-induced mesothelioma must be non-toxic, tolerable, and effective in interfering in asbestos-induced carcinogenesis. LGM2605 reduced asbestos-induced ROS generation, cytotoxicity, and markers of oxidative stress in murine peritoneal macrophages and may impede the asbestos-induced oxidative signaling cascade on the way to malignancy. Importantly, the ability of LGM2605 to interfere in multiple molecular pathways (boosting antioxidant defenses and scavenging free radicals) provides strong evidence for its potential usefulness as a chemopreventive agent in asbestos-induced mesothelioma. The current study provides encouraging findings that support the further evaluation of LGM2605 in chronic, *in vivo* models of MM.

## Abbreviations

8-IsoP	8-iso Prostaglandin F2a
ARE	antioxidant response element
CTL	control
ED	enterodiol
EL	enterolactone
ELISA	Enzyme-linked immunosorbent assays
FLC	Flaxseed Lignan Component
GSTM1	glutathione *S*-transferase mu 1
HO-1	heme oxygenase-1
IACUC	Institutional Animal Care and Use Committee
IP	intraperitoneal
KEAP1	kelch-like ECH-associated protein 1
LDH	lactate dehydrogenase
MF	macrophages
MDA	malondialdehyde
MDA-TBA	malondialdehyde-thiobarbituric acid
MM	Malignant Mesothelioma
Nrf2	nuclear factor (erythroid-derived 2)-like 2
Nqo1	NADPH: quinone oxidoreductase-1
PBS	phosphate-buffered saline
PL	peritoneal lavage
PLF	peritoneal lavage fluid
qPCR	quantitative polymerase chain reaction
RNS	reactive nitrogen species
ROS	reactive oxygen species
SDG	secoisolariciresinol diglucoside
UICC	Union Internationale Contre le Cancer
WBC	white blood cells
